# Integrated Approach Reveals Role of Mitochondrial Germ-Line Mutation F18L in Respiratory Chain, Oxidative Alterations, Drug Sensitivity, and Patient Prognosis in Glioblastoma

**DOI:** 10.3390/ijms20133364

**Published:** 2019-07-09

**Authors:** Kathleen Keatley, Samuel Stromei-Cleroux, Tammy Wiltshire, Nina Rajala, Gary Burton, William V. Holt, D. Timothy J. Littlewood, Andrew G. Briscoe, Josephine Jung, Keyoumars Ashkan, Simon J. Heales, Geoffrey J. Pilkington, Brigitte Meunier, John E. McGeehan, Iain P. Hargreaves, Rhiannon E. McGeehan

**Affiliations:** 1 Brain Tumour Research Centre, Institute of Biological and Biomedical Sciences, School of Pharmacy and Biomedical Sciences, University of Portsmouth, Portsmouth PO1 2DT, UK; 2 Centre for Enzyme Innovation, Institute of Biological and Biomedical Sciences, School of Biological Sciences, University of Portsmouth, Portsmouth PO1 2DY, UK; 3 Institute of Cosmology and Gravitation, University of Portsmouth, Portsmouth PO1 3FX, UK; 4 Academic Unit of Reproductive and Developmental Medicine, University of Sheffield, Sheffield S10 2SF, UK; 5 Department of Life Sciences, Natural History Museum, London SW7 5BD, UK; 6 Core Research Laboratories, Natural History Museum, London SW7 5BD, UK; 7 Department of Neurosurgery, Kings College Hospital, London SE5 9RS, UK; 8 Neurometabolic Unit, National Hospital for Neurology, London WC1N 3BG, UK; 9 Department of Chemical Pathology, Great Ormond Street Hospital for Children NHS Foundation Trust, London WC1N 3JH, UK; 10 UCL Great Ormond Street Institute of Child Health, London WC1N 1EH, UK; 11 Institute for Integrative Biology of the Cell, 91190 Gif-sur-Yvette, France; 12 School of Pharmacy and Biomolecular Sciences, Liverpool John Moores University, Liverpool L3 3AF, UK

**Keywords:** glioblastoma, mtDNA, mutation, mitochondria, OXPHOS, ROS, CoQ10, prognosis, clomipramine

## Abstract

Glioblastoma is the most common and malignant primary brain tumour in adults, with a dismal prognosis. This is partly due to considerable inter- and intra-tumour heterogeneity. Changes in the cellular energy-producing mitochondrial respiratory chain complex (MRC) activities are a hallmark of glioblastoma relative to the normal brain, and associate with differential survival outcomes. Targeting MRC complexes with drugs can also facilitate anti-glioblastoma activity. Whether mutations in the mitochondrial DNA (mtDNA) that encode several components of the MRC contribute to these phenomena remains underexplored. We identified a germ-line mtDNA mutation (m. 14798T > C), enriched in glioblastoma relative to healthy controls, that causes an amino acid substitution F18L within the core mtDNA-encoded cytochrome b subunit of MRC complex III. F18L is predicted to alter corresponding complex III activity, and sensitivity to complex III-targeting drugs. This could in turn alter reactive oxygen species (ROS) production, cell behaviour and, consequently, patient outcomes. Here we show that, despite a heterogeneous mitochondrial background in adult glioblastoma patient biopsy-derived cell cultures, the F18L substitution associates with alterations in individual MRC complex activities, in particular a 75% increase in MRC complex II_III activity, and a 34% reduction in CoQ10, the natural substrate for MRC complex III, levels. Downstream characterisation of an F18L-carrier revealed an 87% increase in intra-cellular ROS, an altered cellular distribution of mitochondrial-specific ROS, and a 64% increased sensitivity to clomipramine, a repurposed MRC complex III-targeting drug. In patients, F18L-carriers that received the current standard of care treatment had a poorer prognosis than non-carriers (373 days vs. 415 days, respectively). Single germ-line mitochondrial mutations could predispose individuals to differential prognoses, and sensitivity to mitochondrial targeted drugs. Thus, F18L, which is present in blood could serve as a useful non-invasive biomarker for the stratification of patients into prognostically relevant groups, one of which requires a lower dose of clomipramine to achieve clinical effect, thus minimising side-effects.

## 1. Introduction

Glioblastoma (GBM; WHO grade IV) is the most malignant and common primary brain tumour in adults [1]. The median survival for glioblastoma patients well enough to receive the standard of care treatment is just 14.6 months post-diagnosis [2]. Effective treatment is hampered by numerous factors, including diverse underlying molecular biology between different tumours and even within the same tumour. This is further impacted by the current reliance on treatments that involve resection, radiotherapy and DNA alkylating agents including temozolomide (TMZ). Research over the last decade has revealed the isocitrate dehydrogenase 1 (IDH1) R132 mutation that predicts a better overall survival rate, and the methylation status of O6-methylguanin-DNA-methyltransferase (MGMT) that predicts a better TMZ response. Additionally, the existence of six main subgroups, classified on the basis of cellular origin, anatomical compartment, various molecular alterations, and transcriptomic signatures, linked to different prognoses or responsiveness to therapies have also been revealed [3,4,5]. Despite these discoveries, the grim reality is that markers that successfully inform treatment options remain limited with little improvement in survival (of more than about a month) being achieved over the last 40 years. 

As mitochondrial alterations are a hallmark of glioblastoma compared to normal brain cells [6,7], and indeed between different glioblastoma, researchers have been investigating their involvement in tumour development, progression, and drug sensitivity, with the aim of identifying new predictive and prognostic markers that inform treatment options, and drug development.

Mitochondria are cellular organelles with a whole host of features reminiscent of their alpha-proteobacterial origin. One such feature includes a double-stranded super-coiled genome, mitochondrial DNA (mtDNA), which has 38 regions. Among the regions, 13 genes encode subunits of the oxidative phosphorylation (OXPHOS) system, while 2 rRNAs and 22 tRNAs are involved with the translation of the OXPHOS mRNAs into proteins within the mitoribosomes. Finally, the D-loop serves as the control region for mtDNA transcription/replication initiation through the interaction of a series of regulatory elements with numerous nuclear DNA-encoded factors that are imported into the mitochondria. 

The OXPHOS system, which is comprised of a series of individual multi-protein complexes, I-IV (the mitochondrial respiratory chain, or MRC) and V (the ATP synthase), is housed within the mitochondrial inner membrane. In addition to the mitochondrial encoded subunits, ~ 70 nuclear subunits complete this elegant molecular machine, which is responsible for producing most of the ATP for cellular metabolism. In addition to ATP generation, numerous other cellular processes are coupled to the electron transfers and proton translocations of the MRC, including the regulation of nucleotide pools, tricarboxylic acid-cycle flux, one-carbon metabolism and reactive oxygen species (ROS) signaling, calcium transport, NADPH generation, ATP/ADP exchange, protein import, inorganic phosphate transport, mitochondrial membrane potential, and apoptosis (reviewed in) [8]. Given the importance of the OXPHOS system, it is not surprising that genetic aberrations in both mtDNA and nuclear DNA (nDNA) genes that encode its suite of proteins have the propensity to not only affect mitochondrial function, but also to cause, or contribute to, disease (reviewed in) [8]. 

For example, it is well established that mutations in nuclear genes that encode succinate dehydrogenase (SDH, MRC complex II), contribute to the pathology of cancers by altering corresponding MRC complex II activity, and substrate levels. Such alterations in turn can also alter the production of ROS, cell redox state and consequently, activate transcription factors such as HIF1-alpha and FOS-JUN, which can also alter gene expression and stimulate cancer cell proliferation [9,10,11]. Increased ROS levels can also lead to increases in basal levels of oxidative stress, making cancer cells vulnerable to agents that further increase ROS levels, e.g., so-called pro-oxidant drugs. 

Changes in MRC complex I–IV activities have been described in glioblastoma compared to the normal brain [12], and between different glioblastoma samples [13]. Furthermore, changes in MRC complex IV activity have been useful in stratifying patients into subgroups with different survival outcomes [13]. Several drugs that were shown to target and inhibit the MRC complexes IV and III over 50 years ago also have promising anti-glioblastoma activity in pre-clinical models—this includes chlorpromazine an antipsychotic agent [14] and clomipramine a tricyclic antidepressant (TCA [15,16,17,18]), respectively. More recently, another TCA, imipramine, was shown to convey therapeutic benefit to tumour baring animals via increasing autophagy-associated cell death [19].

Although the mtDNA mutation spectrums of glioblastoma differ from those of normal brain, and are highly variable between different glioblastoma samples [20,21], whether differences in mtDNA mutations contribute to the differential mitochondrial activities and cellular behaviours, including response to drugs, observed in glioblastoma, has not been significantly investigated. We hypothesise that mutations in mtDNA genes that encode subunits of the OXPHOS system also have the potential to alter OXPHOS activity, ROS levels and cell redox state, thereby contributing to alterations in glioblastoma cell proliferation, and sensitivity to OXPHOS-targeting drugs. Of particular interest in this regard is an inherited mtDNA mutation, M. 14798T > C, that we have previously identified by means of next generation sequencing of glioblastoma mtDNAs. M. 14798T>C occurs more frequently in glioblastoma patients than in healthy individuals (approximately 31% vs. 9%, respectively), and results in a phenylalanine (F) to leucine (L) amino-acid substitution at position 18 of the core subunit of respiratory complex III; MT-CYB. F18L, and due to its location within the MT-CYB CoQ10 (ubiquinone)/inhibitor binding site known as the Qi-site, it is predicted to cause changes in complex III activity and sensitivity to the Qi-site-targeting drug clomipramine [20]. This in turn, could affect CoQ10 levels, ROS production and cellular proliferation rates, and consequently, patient prognoses.

Consequently, in this study we sought to characterise several MRC-related parameters, including MRC complex III targeted drug sensitivity, in adult glioblastoma patient biopsy-derived cell cultures, to determine whether there was an association with F18L-carriers and non-carriers in vitro. We also conducted a retrospective cohort study of adult glioblastoma patients to determine the prognostic relevance of F18L. Here we show that F18L, through structural and functional alterations to MRC complex III, could predispose individuals to differential prognoses, and sensitivity to mitochondrial targeted drugs that may have applicability in treating glioblastoma.

## 2. Results

### 2.1. MRC Complex Activities and CoQ10 Levels Differ in Glioblastoma Cells Relative to Control

**Figure 1:**
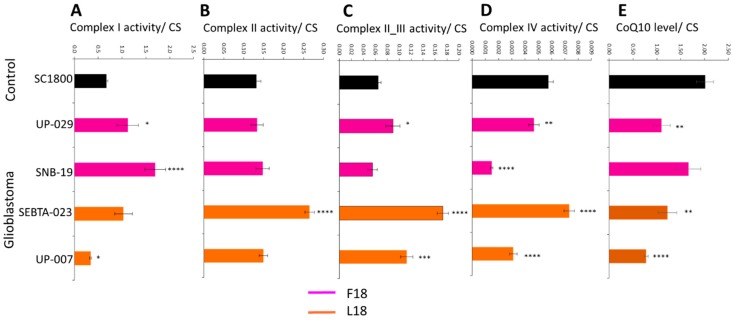
Mitochondrial respiratory chain complex activities and CoQ10 levels in primary adult glioblastoma and normal adult astrocyte cell cultures. (**A**) complex I (NADH-ubiquinone oxidoreductase), (**B**) complex II (succinate-ubiquinone oxidoreductase), (**C**) complex II_III (succinate-dehydrogenase cytochrome c reductase), (**D**) complex IV (cytochrome c oxidase) activity were measured spectrophotometrically, and (**E**) CoQ10 levels were measured via reverse phase HPLC. Data are expressed as mean respiratory chain complex activity or CoQ10 level relative to citrate synthase (CS) activity ± standard error mean (SEM). For respiratory chain complex activities, all cell cultures were measured in triplicate on three independent occasions to give *n* = 9 in each case, and for CoQ10 levels, all cell cultures were measured in triplicate. Asterisks highlight statistically significant differences between adult glioblastoma that are either carriers of F18L (SEBTA-023 and UP-007, orange) or non-carriers (UP-029 and SNB-19, magenta) relative to adult astrocyte (SC1800, black) cell cultures (where *, **, ***, and **** is *p* < 0.05, 0.005, 0.0005, and 0.00005, respectively).

The differences in specific MRC activities compared to normal brain controls were highly dependent on the glioblastoma cell cultures investigated (for additional patient and cell culture characteristics; Table S1A). The activity of MRC complex I was elevated in two cultures (UP-029 and SNB-19, *p* < 0.05 and *p* < 0.00005, respectively), reduced in one other (UP-007, *p* < 0.05), and was similar in the remaining culture (SEBTA-023) relative to the normal brain control culture (SC1800) (Figure 1A). MRC complex II activities of glioblastoma cultures (UP-029, SNB-19, and UP-007) were in general similar to that of the control (SC1800), the exception being SEBTA-023 which had elevated activity (*p* < 0.00005) (Figure 1B). MRC complex II/III activity was similar for SNB-19 compared to the control (SC1800), but was elevated in UP-029, SEBTA-023 and UP-007 (*p* < 0.05, *p* < 0.00005, and *p* < 0.005, respectively) (Figure 1C). The activities of MRC complex IV were either lower (UP-029 and SNB-19, *p* < 0.005 and *p* < 0.00005, respectively) or higher (SEBTA-023 and UP-007, *p* < 0.00005) in glioblastoma cultures relative to the control (SC1800) (Figure 1D). The CoQ10 levels expressed as a ratio to citrate synthase (CS) activity were reduced in glioblastoma cultures relative to the control (see UP-029 (*p* < 0.005), SEBTA-023 (*p* < 0.005), and UP-007 (*p* < 0.00005)). One exception to this, however, was SNB-19, the glioblastoma cell culture bought from a commercial supplier, which had similar CoQ10 levels expressed as a ratio to CS activity compared to the control (Figure 1E).

### 2.2. Individual Analysis of MRC Complexes and CoQ10 in Glioblastoma Cells Reveals Biological Variability

Due to each glioblastoma cell line exhibiting a unique MRC activity profile relative to control (Figure 1), we chose to investigate the biological variability among the glioblastoma cell cultures. Univariate analyses revealed that the overall effect of individual glioblastoma cultures was significant for each of the complexes I, II, II_III and IV (*p* < 0.0005, *p* < 0.000005, *p* < 0.000005, and *p* < 0.000005, respectively) and CoQ10 levels (*p* < 0.007). Furthermore, significant associations between F18L status of the glioblastoma cultures and individual MRC activities was observed, particularly for complex II_III activity. The complex I activity of F18L-carriers was significantly lower compared to non-carriers (0.68 ± 0.12 vs. 1.41 ± 0.17, *p* < 0.005), while the complex II, II_III and IV activities of F18L-carriers were all significantly higher compared to non-carriers (0.21 ± 0.02 vs. 0.14 ± 0.02, *p* < 0.005; 0.14 ± 0.01 vs. 0.08 ± 0.009, *p* < 0.000005; and, 0.006 ± 0.0006 vs. 0.004 ± 0.0005, *p* < 0.005, respectively). Although the CoQ10 levels expressed as a ratio to CS activity of F18L-carriers was lower (1.00 ± 0.14) than in non-carriers (1.38 ± 0.19), the effect only approached statistical significance (*p* = 0.13).

### 2.3. Simultaneous Analysis of Mitochondrial Parameters Reveals Novel Associations 

**Figure 2:**
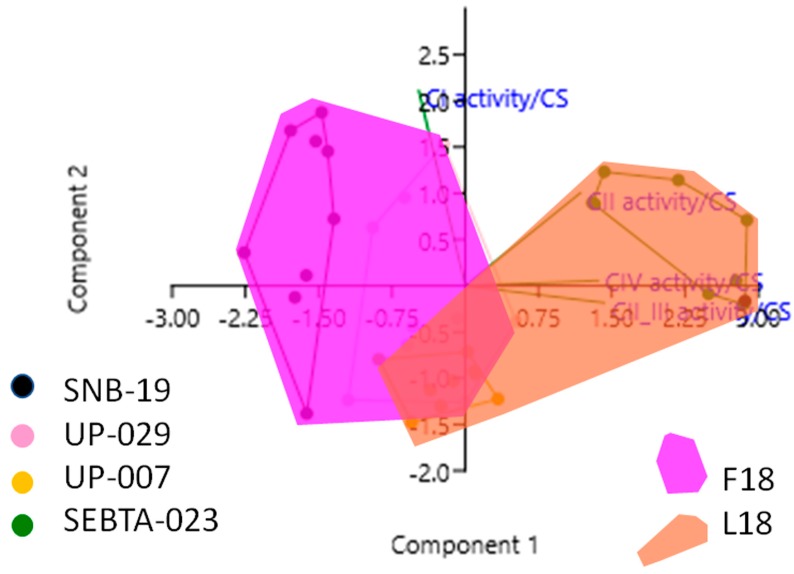
Principal Component Analysis (PCA) plot showing the multivariate variation among four cell lines examined in terms of their complex I, II, II_III and IV activities (expressed relative to citrate synthase (CS) activity in each case). Vectors (green lines) indicate the direction and power of each complex activity dataset relative to the overall distribution. Coloured dots delineate the four cell lines, while the pink and the orange shaded areas highlight the two groups identified in this study, that correlate with the F18L-carriers and non-carriers, respectively. The first two principal axes shown explained 83% of the variance observed among the data.

Although the univariate analyses above revealed associations between individual MRC complex activities and the F18L status of glioblastoma cell cultures, given the biological variability among the glioblastoma cell cultures in terms of their individual MRC complex activities, a series of multivariate analyses were used to investigate all the variables: F18L status, individual MRC complex activities, and CoQ10 levels, simultaneously. Consistent with the univariate analyses, multiple regression analyses revealed that all MRC complex activities were significantly different between F18L-carriers and non-carriers (Table S2A). However, of all the MRC complexes, complex II_III activity was found to be the variable with the most discriminatory power between the F18L-carriers and non-carriers (increased, *p* < 0.000005), followed by complex IV activity (increased, *p* < 0.005), complex I activity (decreased, *p* < 0.002), and then complex II activity (increased, *p* < 0.002). Principal component analysis revealed that 83% of the variance from combining the individual MRC complex activity data from all the glioblastoma cell cultures could be deconstructed into two principal components (PC1 and PC2), and when these data were plotted in two dimensions, they formed two distinct subgroups related to the presence or absence of F18L (Figure 2). Discriminant function analyses revealed that 86.1% of observations can be correctly classified into the F18L-carrier and non-carrier sub-groups, highlighting the potential of F18L to predict the MRC complex activity features of glioblastoma cultures (Table S2B).

**Figure 3:**
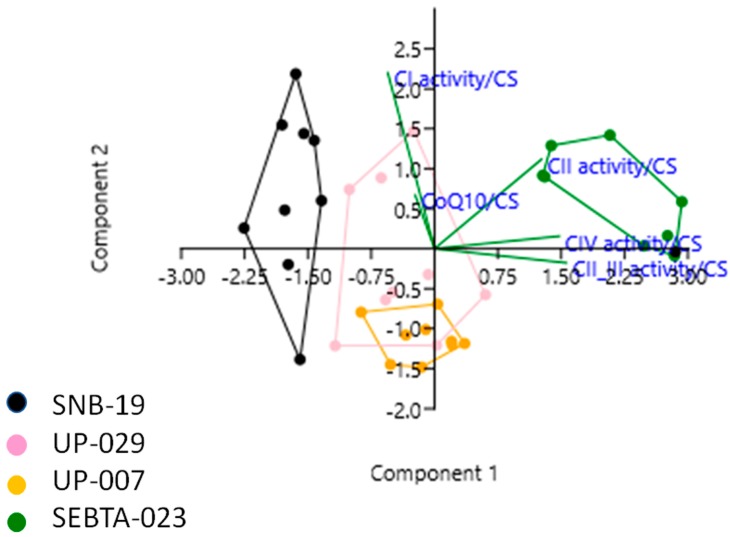
Principal Component Analysis (PCA) plot showing the multivariate variation among four cell lines examined in terms of their complex I, II, II_III and IV activities, and CoQ10 levels (expressed relative to citrate synthase (CS) activity in each case). Vectors (green lines) indicate the direction and power of each complex activity dataset relative to the overall distribution. Coloured dots delineate the four cell lines. The first two principal axes shown explained 78% of the variance observed among the data.

Intriguingly, when individual MRC complex activity data were also combined with the CoQ10 level data, and also analysed by PCA, further associations were identified. Specifically, positive correlations were observed between MRC complex I activity and CoQ10 level, MRC complex II and IV activities, MRC complex III and IV activities. In addition, a negative correlation was observed between MRC complex II_III activity and CoQ10 level (Figure 3, Table S2C). 

### 2.4. F18L-Carriers Have Poorer Prognosis

**Figure 4:**
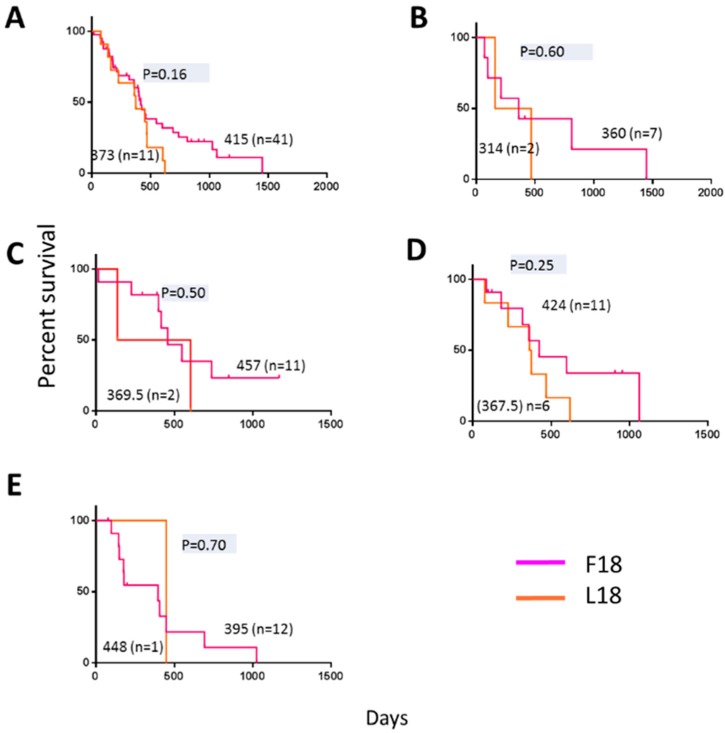
Kaplan-Meier estimates of overall survival in 52 primary adult glioblastoma patients present in the cancer genome atlas (TCGA) database previously assigned a gene expression subtype [5] and stratified based on F18L status (this study). The overall median survival for F18L-carriers are shown in magenta and non-carriers in orange for (**A**) all subtypes analysed together, and (**B**) classical, (**C**) neural, (**D**) mesenchymal, and (**E**) pro-neural subtypes. Horizontal dashes denote censored points, and median overall survival for all groups (no brackets), the number of patients in each group (brackets), and *p*-values (grey) are also indicated.

Previously, elevated complex III and IV activities have been observed in temozolomide resistant glioblastoma cells, leading to the hypothesis that both elevated complex III and IV activity could be predictors of poor outcome [22]. Subsequently, elevated complex IV activity has been confirmed as a predictor of poor outcome [13]. Consequently, we investigated if the presence of F18L, a potential marker of elevated complex III activity, could be of direct prognostic value. For this, 52 primary adult glioblastoma tissues with matched genomic and clinical data previously gathered by the cancer genome atlas (TCGA) were also screened for the presence of F18L (for additional glioblastoma patient and tissue characteristics; Table S1B). These glioblastoma tissues had also been previously classified into four clinically relevant gene-expression subtypes termed classical, neural, mesenchymal, and proneural [5]. The overall frequency of F18L-carriers in the TCGA cohort analysed was 22%. Relative to the overall TCGA cohort analysed, some variability in the frequency of F18L was observed among the glioblastoma subtypes, with a slightly increased frequency (35%) in the mesenchymal group and a slightly decreased frequency in the proneural group (8%) (Figure 4, Table S1B). Although the samples sizes were too small for the results to be significant, F18L-carriers have a poorer prognosis than non-carriers overall (i.e., when all subtypes were analysed together and then stratified based on the presence or absence of F18L, the median overall survival was 373 days vs. 415 days, respectively) (Figure 4A). Notably, glioblastoma subtype did not affect this result (Figure 4B–E). Parameters such as male skew (64% vs. 76%), MGMT methylation (25% vs. 27%), Karnofsky score (71 vs. 82) and age at diagnosis (64 vs. 59) did not vary between F18L-carriers compared with non-carriers, respectively, suggesting that the presence of F18L could be a valuable independent marker of poor prognosis, although this will require validation using additional cohorts in the future.

### 2.5. F18L-Carriers Have Elevated Oxidative Stress

**Figure 5:**
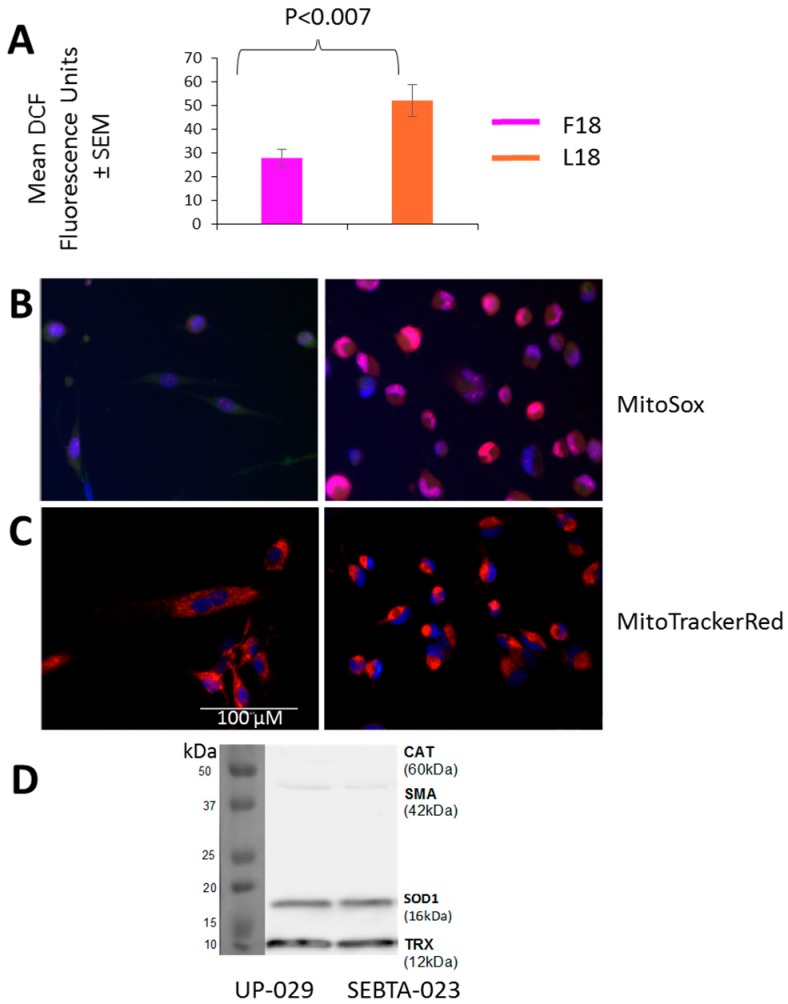
Reactive oxygen species (ROS) production, mitochondrial localisation and antioxidant protein levels in non-carrier UP-029 and F18L-carrier SEBTA-023 primary adult glioblastoma cell cultures. (**A**) Overall intra-cellular levels were determined in cells using 1 mM DCF-DA. After one h, fluorescence was recorded on the BMG LABTECH FLUOstar Omega Plate reader. For each cell culture, 10,000 cells were measured in triplicate on three independent occasions to give an average value ± SEM based on *n* = 9 in each case. (**B**) Localisation of mitochondrial ROS was determined in cells (representative images shown) using 5 μM MitoSOX^TM^ (red). After 30 min, fluorescence was recorded using live cell imaging. (**C**) Distribution of mitochondria was determined in cells (representative images shown) using 500 nM MitoTrackerRed (red). After 30 min, fluorescence was recorded using live cell imaging. Mitochondrial ROS and mitochondria are both shown relative to cell nuclei which were stained with 0.02 mg/mL Hoechst 33342 (blue). (**D**) Representative immunoblot showing the protein levels of (from top to bottom): catalase (CAT), smooth muscle actin (SMA, loading control), superoxide dismutase 1 (SOD1) and thioredoxin (TRX) oxidative defense enzymes in whole cell lysates (20 µg of total protein). All images are set to the same scale, as indicated by the scale bar.

Alterations in MRC complex activities are often accompanied by changes in intra-cellular ROS levels due to the MRC, in particular complex III, being a major site for ROS generation [23]. Additionally, the electron carrier CoQ10 also serves a ROS scavenger [24], and so a difference in levels could also affect intra-cellular ROS levels. Consequently, we sought to measure the overall cellular levels of intra-cellular ROS in a representative F18L-carrier and non-carrier using the vital dye 2′,7′-dichlorofluorescein (DCF) green (which measures a whole range of ROS types, including the superoxide anion produced by mitochondria, which is the precursor of the majority of all other ROS present within the cell). Intra-cellular ROS levels were markedly elevated in F18L-carriers relative to non-carriers (52.16 ± 6.81 vs. 27.9 ± 3.53, *p* < 0.007) (Figure 5A).

Cellular ROS production is compartmentalised and it is therefore important to differentiate between intra-cellular and mitochondrial species. Consequently, mitochondrial ROS were also monitored using MitoSoxRed and live cell imaging. The major difference observed between F18L-carriers and non-carriers was the cellular distribution of mitochondrial ROS, with the F18L-carriers exhibiting a predominantly peri-nuclear localisation and the non-carriers exhibiting a pan-cytoplasmic localisation (Figure 5B). Live cell imaging of the mitochondrial networks with MitoTrackerRed suggests that the differential cellular distributions of mitochondrial ROS observed between F18L-carriers and non-carriers is associated with the redistribution of mitochondria themselves (Figure 5C). Elevated intra-cellular ROS and the redistribution of mitochondria to the peri-nuclear region in F18L-carriers, are both consistent with cells exhibiting signs of elevated oxidative stress [25].

Differences in the protein levels of other ROS scavengers have been observed both between glioblastoma and healthy cells, and between glioblastoma cells derived from different tumours. These differences have previously been linked to variance in patient prognosis (e.g., overall survival) and the chemosensitivity of glioblastoma cells in vitro [26,27,28,29,30], consequently we wanted to check differences in antioxidant levels were not responsible for the changes in ROS observed between F18L-carriers and non-carriers. We confirmed that the protein levels of the key ROS scavengers, catalase, SOD1 and TRX, were unaltered in F18L-carriers (Figure 5D).

### 2.6. F18L-Carriers Are More Sensitive to Clomipramine

**Figure 6:**
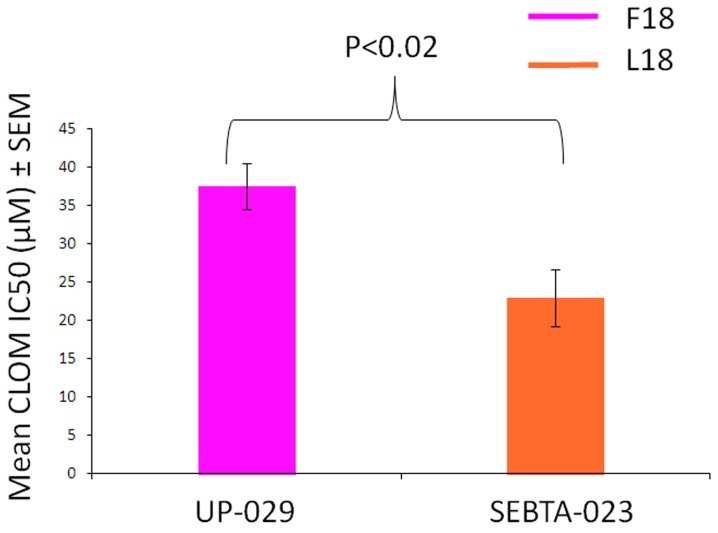
Clomipramine sensitivity of both non-carrier UP-029 and F18L-carrier SEBTA-023 primary adult glioblastoma cell cultures over a period of 72 h. The proliferation of cells measured using the RealTime-Glo™ MT Cell Viability Assay (Promega) without clomipramine (0 µM) and different doses of clomipramine (range 5–50 µM) was monitored by measuring cumulative luminescence generated by a pro-substrate being converted into a substrate in viable cells at 1, 3, 6, 24, 48 and 72 h. The proliferation of cells treated with clomipramine were then normalised to the without clomipramine control at each time point. The normalised viability curves were then used to calculate the clomipramine IC50 (µM) values for each cell line (all time points combined). Each cell line, at each time point and concentration (including 0 µM), was measured in triplicate on three independent occasions giving a mean value ± SEM based on *n* = 9 in each case.

Clomipramine is a tricyclic antidepressant drug that is effective at killing glioblastoma cells via mitochondrially-mediated apoptosis [15,16,17,18]. It has also been shown that clomipramine inhibits MRC complex III activity in isolated rat, bovine and yeast mitochondria (reviewed in [31]). These studies raised the possibility that clomipramine sensitivity may be affected by the presence of mtDNA mutations that influence complex III properties. Further support for this hypothesis has been obtained through yeast studies, where single mtDNA mutations introduced into the yeast Qi-site either increase or decrease complex III sensitivity to clomipramine [31]. For example, yeast complex III that contains an isoleucine at position 17 of MT-CYB and is analogous to the MT-CYB of an F18L-carrier in glioblastoma, is 2-fold more sensitive to clomipramine compared to yeast complex III that contains phenylalanine at position 17 of MT-CYB, which is analogous to the MT-CYB of a non-carrier in humans [31]. Consequently, we sought to determine whether F18L-carrier glioblastoma cells were more sensitive to clomipramine-growth inhibition compared to non-carriers. Viability assays over 72 h with different doses of clomipramine (0–50 µM) confirmed the growth inhibitory effect of clomipramine on all glioblastoma cells, as well as the amount of clomipramine required to inhibit the growth by half (relative to the 0 µM control), at 1, 3, 6, 24, 48 and 72 h. Over the 72 h period, F18L-carrier cells yielded a lower IC50 value, i.e., were x1.64-fold more sensitive to clomipramine than non-carrier cells (mean IC50 µM 22.9 ± 3.75 vs. 37.51 ± 3.02, *p* < 0.02) (Figure 6). These data suggest an association between F18L and enhanced complex III-targeted drug sensitivity in glioblastoma cells.

**Figure 7:**
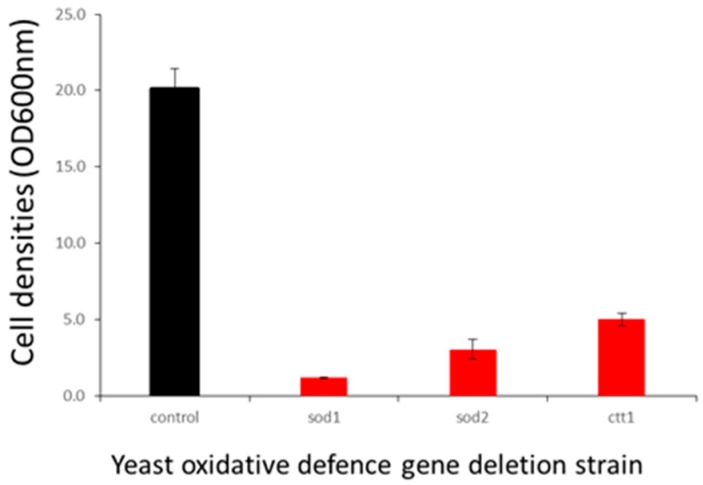
Effect of oxidative defense gene deletion on clomipramine sensitivity. Control strains (BY4742) and their derived deletion mutants were grown in YPEth (respiratory medium) in absence (black) or presence of 200 µM (red) clomipramine with vigorous shaking for good aeration. Cell densities (OD600 nm) were estimated after three days of growth.

The enhanced clomipramine sensitivity of F18L-carriers relative to non-carriers could also be due to elevated oxidative stress. Using the yeast model, we checked the inhibitory effect of clomipramine on the respiratory growth of a series of mutants with deletions of 17 genes involved in oxidative stress defense, a full list of the genes tested, the role and subcellular localization of the gene products are listed in Table S3. The control strain and its derived deletion mutants were cultured in respiratory medium in the presence or absence of 200 µM clomipramine and the cell density (estimated by the OD at 600 nm) was measured after three days of incubation at 28 °C with vigorous shaking for good aeration. Of the mutants tested, three displayed increased sensitivity to clomipramine (in order of severity): Δsod1, lacking the mainly cytosolic superoxide dismutase, Δsod2, lacking the mitochondrial superoxide dismutase and Δctt1, lacking cytosolic catalase (Figure 7). The data suggest that clomipramine treatment results in oxidative stress, as it is the mutant cells that have their first-line of anti-oxidant defenses compromised that are highly sensitive to the drug.

We observed that glioblastoma cells that are F18L-carriers have elevated oxidative stress and increased growth sensitivity to clomipramine. It could be suggested that in these cells, the clomipramine-induced oxidative stress is enhanced by the basal oxidative stress. The differential sensitivity of glioblastoma cells might thus be explained by a combination of increased complex III inhibition and enhanced oxidative stress in F18L-carriers.

## 3. Discussion

There is considerable room for improving current glioblastoma management regimens and treatments. Patients and families feel confused and disillusioned by the knowledge of multiple molecular prognostic and predictive markers presented to them after diagnosis as no matter their molecular background, they still tend to receive the current standard of care treatment.

Inter-tumoural mitochondrial functional heterogeneity is commonplace in glioblastoma [12,13]. One possibility is that the differences observed represent an adaptive response by the mitochondria to well-known oncogenic pathways and the factors that influence them and lead to their stabilisation. One example of such an adaptation includes the HIF/hypoxia pathway, where hypoxic microenvironments can lead to the stable expression of HIF1 and 2 alpha, which in turn bind to the promoters and consequently regulate the expression of numerous genes and a more glycolytic and proliferative phenotype (reviewed in) [32]. An alternative possibility, however, is that the mitochondrial functional differences observed are a consequence of the specific mtDNA sequence variations present within the glioblastoma. Indeed, mtDNA sequence mutation spectra also vary inter-tumourally in glioblastoma [20,21], and these could contribute to glioblastoma development and progression, including in response to treatment. However, due to limited mtDNA genotype-cellular phenotype studies, a deeper understanding is required, as to how mtDNA sequence variations associate with mitochondrial functional abnormalities. Possible drivers include changes in individual mitochondrial respiratory chain activities and other related parameters, such as ROS production, affecting oncogenic pathways, subsequent response to treatments, and ultimately patient survival.

**Figure 8:**
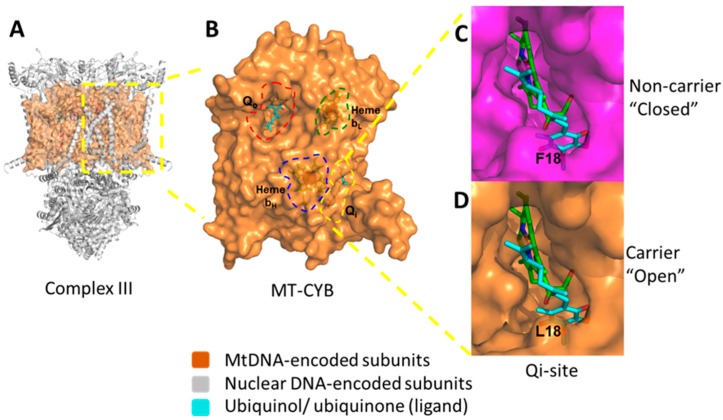
Structural consequences of the inherited homoplasmic mutation F18L found frequently in the glioblastoma complex III mitochondrial protein; MT-CYB. (**A**) The dimeric structure of complex III with the nuclear-encoded subunits shown as ribbons (grey) and mitochondrial-encoded subunit, MT-CYB, shown as a space filling model (orange). (**B**) Key functional sites within MT-CYB are highlighted; the Qo-site which binds ubiquinol (cyan sticks, red dashed lines), the Qi-site which binds CoQ10 (also known as ubiquinone) (cyan sticks, yellow dashed lines), heme bL (orange sticks, green dashed lines) and heme bH (orange sticks, blue dashed lines). (**C**) The “closed” Qi-site pocket (top, magenta) present in non-carriers adult glioblastoma cell lines UP-029 and SNB19. (**D**) The “open” Qi-site pocket (bottom, orange) present in F18L-carriers adult glioblastoma cell lines SEBTA-023 and UP-007. Bound ubiquinone is shown as sticks (cyan) in both cases. F18L, through the changes to the local structural environment shown, is predicted to lead to alternative modes of CoQ10 association/disassociation. This in turn could affect OXPHOS (particularly complex III) activity, ROS production, basal levels of oxidative stress, and consequently, cell behaviours (e.g., proliferation rate both with and without complex III-targeted drugs like clomipramine), and patient prognosis.

Here we investigated specific associations between the presence of germ-line mtDNA mutation F18L, previously found to be enriched in glioblastoma relative to healthy controls [20], and changes in multiple mitochondrial properties within adult glioblastoma cell lines in vitro. This study employed a systematic analysis of individual MRC complex activities, CoQ10 levels, intracellular and mitochondrial ROS levels, and sensitivity to the MRC complex III-targeting drug, clomipramine, and revealed complex heterogeneity between glioblastoma cell lines. We observed associations between the F18L status of glioblastoma cell lines, and MRC complex activities (particularly complex II_III activity), CoQ10 levels, and ROS levels, despite the heterogeneous background of OXPHOS activity. Taking these associations together with the predicted effects of F18L on the structure and function of corresponding complex III (Figure 8), we propose that single germ-line mtDNA variations like F18L could mechanistically contribute to the landscape of OXPHOS heterogeneity observed in glioblastoma.

There are a number of approaches that can be employed to identify whether an mtDNA mutation contributes to cellular phenotypes. One is to demonstrate that tumour cell mitochondria containing a mutation have a corresponding mitochondrial defect, another is to observe whether the transfer of tumour cell mitochondria/ mtDNA onto a wild-type background confers an associated mitochondrial defect and phenotype, and another is to introduce the specific mtDNA mutation concerned into yeast cells, this approach is not yet possible in mammalian cells. MtDNA mutations have been shown to play a role in tumourigenesis in cancers other than the brain, e.g., cell lines that harbor a ND5 gene mutation found in colon and renal adenocarcinomas, acute myeloid leukemia and ovarian serous cystadenocarcinoma (reviewed) [33], when compared with wild-type cells, exhibits enhanced colony formation on agar and increased tumour growth when implanted into nude mice (reviewed in) [33]. It has also been shown that when tumour mtDNA containing the ND5 mutation is transferred onto a wild-type background a Warburg like effect (i.e., reliance on glycolysis for energy) is conferred to the cybrids created (reviewed in) [33]. Further, an ND6 gene mutation was found to distinguish hypoxic sensitive from insensitive glioblastoma cells [34]. With the exception of these studies, there have been very few investigations into associations between mtDNA mutations and changes in mitochondrial/ cellular behaviour in glioblastoma.

The observation that F18L associates with a corresponding alteration in complex II_III activity in glioblastoma is consistent with our predicted effects of the F18L mutation on the three-dimensional structure of the coenzyme Q10 (CoQ10)-binding pocket (Qi-site) that lies within the mtDNA-encoded MT-CYB protein, at the heart of complex III [20]. The Qi-site has evolved to make intimate interactions with CoQ10 to action binding, redox reaction, and dissociation. Previously, we have also used an in silico docking approach to model the effect of F18L and wild-type MT-CYB on these interactions, and consequently on CoQ10 binding [20]. We predicted the structure of the binding cavity is opened up by the loss of a bulky aromatic group leading to alternative ubiquinone binding modes in F18L relative to wild-type ([20], Figure 8). We further predicted that these additional modes lead to increased interactions and therefore affinity between the Qi-site and ubiquinone, affecting the crucial balance of association and dissociation of ubiquinone, likely facilitating redox reaction, and consequently, increased complex III activity, thus providing a potential molecular basis for the observed phenotypes.

The presence of F18L correlating with changes in ROS in glioblastoma is also intriguing. Previously, elevated complex III activity, reflecting a more OXPHOS dependent, and efficient and less leaky MRC, has been associated with elevated complex IV activity and, decreased ROS levels in glioblastoma cells [35]. While we observe the same positive correlation between complex III and IV, we observe elevated, rather than reduced, ROS levels in F18L-carriers relative to non-carriers. This apparent paradox could be due to differences in the specific models investigated. [35] observed a parallel increase in both antioxidant enzymes and complex III and IV activity, which would attenuate ROS levels, while we observed no difference in antioxidant defense enzymes, which may lead to elevated ROS levels in F18L-carriers. While the mechanism by which heightened ROS levels can be observed in concert with heightened complex III and IV activity remains to be elucidated, the elevated ROS levels observed in F18L-carriers could be responsible, at least in part, to: (a) the decreased complex I activity through the oxidative damage to complex I, and (b) the slight decrease in CoQ10 levels through the oxidative damage and degradation of CoQ10 [36]. The latter could also reduce antioxidant defenses of the F18L-carriers (CoQ10 has antioxidant activity), leading to further ROS production, and additional complex I damage. So, reduced CoQ10 levels could also be a source of the elevated ROS observed in the F18L-carriers. As MRC-linked ROS are also major components of cell signaling pathways, some of the effects on MRC complex activity and CoQ10 levels may also be secondary to the mutation F18L, since ROS may also effect the expression of molecules such as IMP2 and COXIV-1 (which can regulate OXPHOS activity, [35,36,37], respectively), as well as those involved in CoQ10 biosynthesis [38]. Where CoQ10 levels are reduced (e.g., slightly between F18L-carriers and non-carriers, and in general between glioblastoma and normal brain cells), supplementation with exogenous CoQ10 or its analogues may be beneficial, restoring MRC complex activities to ‘normal levels’. Indeed, it has been demonstrated that exogenously administered CoQ10 can be incorporated into the mitochondria (reviewed in) [39]. It has also been shown to improve disease in human and animal studies in situations where there is genetic CoQ10 deficiency, cardiac failure, Parkinson’s disease, Alzheimer’s disease, Friedreich’s ataxia and ageing, possibly via enhancement of electron transfer efficiencies and attenuating damage caused by ROS (reviewed in) [39].

F18L-carriers have a worse prognosis than non-carriers. Intriguingly, a role for ancient adaptive mtDNA variants, including F18L, through a mechanism not unlike that described here, in affecting predisposition to degenerative diseases and ageing has been proposed previously [40]. However, this is the first time a single mtDNA mutation has been linked to glioblastoma patient outcome, and it raises the possibility that other mtDNA mutations (or combinations of mtDNA and nuclear DNA mutations) may be instrumental in the design of enhanced combinatorial prognostic markers. This has been demonstrated recently in prostate cancer, where certain mtDNA mutations that co-occur with the MYC oncogene jointly associate with patient survival [41]. Validating the prognostic relevance of F18L and other mtDNA mutations in additional cohorts is the subject of ongoing work.

F18L-carriers in vitro are more sensitive to clomipramine. While it has been shown that (a) glioblastoma cells containing an ND5 mutation or an ND6 mutation are more resistant to rotenone (a Complex I inhibitor) and to adriamycin (a chemotherapeutic drug activated by Complex I redox signaling,) [34], respectively, and (b) yeast complex III engineered to contain an analogue of F18L is more sensitive to clomipramine [31], this is the first account of human cells containing F18L showing enhanced clomipramine sensitivity. This approximately 2-fold enhancement of clomipramine sensitivity in yeast and mammalian cells with F18L (or its equivalent) could also be due to the predicted effects of the F18L mutation on the local structural environment of the Qi-site (Figure 8). Like for ubiquinone, we previously used an in silico docking approach to model the effect of F18L and wild-type MT-CYB on clomipramine-Qi-site interactions, and consequently on clomipramine binding. We predicted the opening up of the Qi-site by F18L creates more space for clomipramine binding, which would lead to increased complex III sensitivity and, in turn cell growth sensitivity [20]. However, the yeast anti-oxidant defense knock-outs, specifically SOD1, SOD2 and catalase which are markedly more sensitive to clomipramine sensitivity than wild-type, suggest the heightened intracellular and mitochondrial ROS, F18L mediated or otherwise, could also play a role in clomipramine sensitivity.

Taken together, our observations provide new insights into how mtDNA sequence variations, like F18L, contribute to heterogeneous landscape of glioblastoma. Given the long-term use of TCAs like clomipramine has been anecdotally linked with a decreased incidence of gliomas in patients [42], and have been shown to facilitate anti-glioblastoma activity in pre-clinical models [15,16,17,18,19], it would seem TCAs may have applicability for treating glioblastoma. Should there be a TCA (particularly clomipramine) clinical trial, it is an exciting possibility that F18L status, determinable relatively non-invasively via a simple genetic blood test, could be used as a prospective inclusion criterion. This would allow the usefulness of F18L for stratifying patients into clinically effective groups to be validated. It remains to be seen what the net benefit of F18L for patients would be given it may decrease patient survival under current treatment regimens on the one hand, but increase the clinical effect of clomipramine, on the other.

## 4. Materials and Methods

All reagents, materials and equipment were purchased from Sigma-Aldrich^®^ (Gillingham, UK) and steps carried out at room temperature, unless stated otherwise within the text below or Supplementary Information files.

### 4.1. Patient-Derived Glioblastoma Cell Cultures

Human adult glioblastoma biopsy-derived primary cell cultures: UP-029, SEBTA-023 and UP-007, were obtained from patients from Kings College Hospital, London, under ethics permission (REC reference number: 11/SC/0048, 29 August 2018); 1 human adult glioblastoma primary cell culture was obtained commercially: SNB19; and finally, 1 human adult nonneoplastic astrocyte cell line derived from the cerebral cortex (control) was obtained commercially: SC1800, were used for the F18L mutation identification, and mitochondrial and cellular functional assays (for additional patient and cell line characteristics; Table S1A). Details of the establishment and maintenance of cell cultures are described in the Supplementary Materials and Methods.

### 4.2. Patient-Derived Glioblastoma Tissues

Glioblastoma tissues previously gathered by the cancer genome atlas (TCGA) and classified into clinically relevant gene-expression subtypes [5] were also screened for the presence of the F18L mutation. Only primary adult glioblastoma patients, and those with at least one read covering the nucleotide region encoding F18L were considered. Consequently, just 52/202 of the patients in the original study, which contained several lower grade gliomas, were included in this retrospective cohort overall survival study investigating the prognostic significance of F18L. Overall survival was defined as the time from diagnosis (in days) to death. Time from diagnosis (in days) to last follow up was used for including and censoring individuals that were recorded as alive at the end of the study period. Among the cohort analysed, there was a bias for male patients (38/52) and an unmethylated MGMT genotype (38/52), Karnofsky scores were variable (range 40–100), and age at diagnosis was also variable (range 31–87) (for additional patient and tissue characteristics; Table S1B).

### 4.3. F18L Mutation Identification

For the cell lines, mitochondrial mutation data was collected from UP-029, SNB19 and SC1800 previously using long PCR combined with next generation sequencing [4]. For SEBTA-023 and UP-007, new mitochondrial mutation data was collected using the same approach (Table S1A). Briefly, total DNA was isolated from the cell pellets and amplified by long-PCR using human mtDNA-specific primers CytbF and HumanLongR (Amplicon 1) or HumanLongF and CytbR (Amplicon 2) to yield two halves of the mitochondrial genome in each case, which were then resolved by agarose gel electrophoresis, purified and quantified. Amplicon 1 and 2 from each sample were pooled (0.65 nM each) and 1 ng of DNA from each pool was used to construct 12 Nextera XT fragment libraries (Illumina, Cambridge, UK), which were combined, sequenced with paired-end 2 X 150 reads on the Illumina MiSeq system (Department of Biochemistry, University of Cambridge, London, UK). The Illumina Resequencing Workflow [43] was used to align and compare reads and identify mutations relative to the rCRS. Alignments were performed using Burrows-Wheeler Aligner (BWA [44,45] and the F18L mutation were identified from the alignments using GATK Unified Genotyper [46]. The presence of mutations was further confirmed by visually inspecting the full alignments using Tablet [47]. Consensus mitochondrial genomes were annotated using MacVector v.12.7 (MacVector Inc., Cambridge, UK), using the auto-annotate function with the rCRS as reference and applying default parameters; this method recognized the majority of features for each genome. The inferred positions of all transfer RNA genes were confirmed using tRNA scan SE 1.21 [48] or ARWEN [49], including those not found through auto-annotation. Open reading frames, initiation and termination codons, inferred translated proteins, rRNA genes and control regions were all confirmed by means of multiple alignments performed within Geneious v. 6.1.6 (Biomatters Ltd., Auckland, New Zealand). All features found in the rCRS were confirmed for each new mitochondrial genome. The gene content and arrangement for the two new mitochondrial genomes was the same as for the rCRS. F18L-containing cells included UP-007 and SEBTA-023, while those not containing F18L included UP029, SNB19 and SC1800.

For the glioblastoma tissues analysed as part of the TCGA (Table S1B), total DNA was used to construct whole-exome (WES) or whole-genome (WGS) libraries, which were sequenced either on an Illumina HiSeq 2000 or GA-IIX (76 bp paired-end reads) or GAII or HiSeq (101 bp paired-end reads), respectively. The TCGA’s BWA with Mark Duplicates and Cocleaning workflow was used to align and compare reads relative to GRCh Build 38 (also known as hg38), this also uses the rCRS (for full details [50]). Briefly, alignments were performed using BWA-MEM or BWA-aln depending on the read length [42,43]. Prior to variant calling, alignments were converted to sam format, coordinate sorted and duplicates removed using Samtools (version 1.9, [51]). Local realignment and variant identification was conducted using Platypus (version 0.8.1, [52]). The presence of F18L was inspected further by visualization in Tablet [45]. Only alignments/samples which had the nucleotide region encoding F18L covered by one or more read were considered for the survival analyses.

### 4.4. Measurement of MRC Enzyme and Citrate Synthase Activity and CoQ10 Levels

Measurements of the activities of the MRC enzymes and citrate synthase, together with cellular CoQ10 levels were carried out to a diagnostic level at the Neurometabolic Unit at the National Hospital for Neurology and Neurosurgery, (UCLH) Queen Square. Trypsinised cell pellets were washed in HBSS and frozen immediately at −80 °C until use. For the MRC enzyme activity assays, cells were quickly thawed at 37 °C, vortexed and frozen in liquid nitrogen three times prior to use immediately. NADH-ubiquinone oxidoreductase (complex I, [53]), succinate-ubiquinone oxidoreductase (complex II, [53]), succinate-dehydrogenase cytochrome c reductase (complex II_III, [54]), and cytochrome c oxidase (complex IV, [55]) activities, together with citrate synthase (CS, [56]) activity of the freeze-thawed cells were measured spectrophotometrically according to the method of [57]. Each cell line was analysed in triplicate on three independent occasions.

Total cellular CoQ10 status was determined in cell cultures relative to an internal standard (di-propoxy-CoQ10) by RPHPLC coupled to UV detection at 275 nm according to the method described by [58]. Cells were thawed at 37 °C, and aliquoted for CoQ10, total protein and CS analysis. The internal standard was added to the samples, which were then freeze-thawed a further two times using liquid nitrogen. Hexane/ethanol mixtures were then added to each sample, mixed and centrifuged, and the upper hexane layer collected. This step was repeated once more on the lower layer. The two hexane layers were then evaporated under nitrogen gas, and residue stored at −70 °C until analysed by HPLC. Each cell line was analysed on three independent occasions. All MRC enzyme activities and CoQ10 levels were expressed as a ratio to CS to correct for any potential mitochondrial enrichment of the samples [59].

### 4.5. Intracellular ROS Detection

Glioblastoma cells were seeded at a density of 100,000 cells per 25 cm^2^ flasks in DMEM + 10% FCS without phenol red. After 24 h media was removed from the flasks and cells were gently washed with warmed PBS. Cells were incubated with 1 mM 2′,7′-dichlorofluorescin diacetate (DCFH-DA) solution for 1 h at 37 °C in the dark. Cells were washed with PBS, detached from the flasks and re-suspended in phenol red free DMEM and spun for 5 min at 200 ×g. Cell pellets were re-suspended in 1 mL of DMEM without serum and phenol red and counted using a Countess II FL Automated Cell Counter (Life Technologies, Warrington, UK). A cell suspension of 50,000 cells per mL was created for each cell line and 200 µL was transferred to 3 wells of a 96 well plate to give 10,000 cells per well. Hydrogen peroxide was diluted to 8 mM in clear DMEM without serum and added to independent wells not containing cells as a positive control. Three wells were plated for each cell line. Fluorescence of the samples was then measured using the BMG LABTECH FLUOstar (BMG LABTECH. GmbH, Ortenberg, Germany) Optima plate reader at 480 nm/530 nm at 600 gain. Each cell line was analysed in triplicate on three independent occasions.

### 4.6. Live Cell Imaging of Mitochondrial ROS and Networks

Glioblastoma cells were seeded at a density of 25,000 cells per well of a 24 well plate in DMEM + 10% FCS without phenol red. After 24 h, media was removed from the wells, and the cells were washed three times with clear HBSS with calcium and magnesium. For the analysis of mitochondrial ROS, cells were incubated 5 µM MitoSox red, 200 nM Mitotracker green and 0.02 mg/mL live cell nuclear dye Hoechst 33342 in DMEM without FCS and phenol red for 30 min. For the analysis of mitochondrial networks, cells were incubated with 500 nM Mitotracker red (Thermofisher, Altrincham, UK) and Hoechst 33342 (as described above) in DMEM without FCS and phenol red for 30 min. In both cases, the dyes were removed, the cells washed (as described above) and 500 µL DMEM without FCS and phenol red placed on top of the cells to keep them viable throughout imaging. Cells were imaged using an EVOS auto FL live cell imager (Life Technologies, Warrington, UK), inside an atmospheric chamber set to 37 °C, 5% CO_2_ and 19% O_2_. Fluorescence was measured in the RFP, GFP and DAPI channels for the MTS and Texas Red and DAPI channels for the MTR experiments. Each cell line was analysed in triplicate.

### 4.7. Immunoblotting

Frozen-thawed cell lysates, containing 20 μg total protein were separated by SDS-PAGE using 10% polyacrylamide gels and transferred to a PVDF membrane (BioRad, Watford, UK) for Western blot analysis. Blots were blocked using 5%(*w*/*v*) milk; 10% (*v*/*v*) Tris buffered saline and 0.1% (*v*/*v*) Tween 20 (TBST) for 1 h; probed with rabbit monoclonal oxidative stress defense WB cocktail (#ab179843, Abcam, Cambridge, UK) diluted 1 in 250 in blocking buffer in blocking buffer for 1 h. Blots were subsequently incubated with anti-rabbit IgG HRP conjugate (#W4011; Promega, Southampton, UK), diluted to 0.1 μg/mL^−1^ in blocking buffer for 1 h. After each incubation step, the blot was washed five times for 5 min in TBST. Finally, the blot was incubated for 1 min in Luminata™ forte Western HRP substrate (Merck Millipore, Watford, UK). Positive immunoreactivity in the glioblastoma cell lysates was detected by measuring the chemoluminescence for up to 90 s in the dark using a GBOX detector (SynGene, Cambridge, UK). Each cell lysate was analysed on three independent occasions. Densitometry analysis was conducted on all bands using GeneSnap software (SynGene, Cambridge, UK). Bands were selected and a value of band intensity was calculated after background subtraction.

### 4.8. Analysis of Cell Proliferation with and without Clomipramine

Cells (1250/well) were seeded into 96 well tissue culture plates along with the Real Time Glo reagents (x1 final concentration) followed by 0 (medium only control), 0 (DMSO-vehicle only control), 5, 10, 25 and 50 µM (final concentration) of the MRC complex III targeting drug, clomipramine, all in DMEM + 10% FCS. The Real Time Glo reagents: luciferase and cell-permanent pro-substrate were added to the cells. Viable cells reduce the pro-substrate to a substrate into the surrounding medium and reacts with the luciferase to produce a luminescent signal. As the luciferase and substrates are stable in culture for 72 h, repeated measurements on the same wells can be made up to this point. Consequently, luminescence was measured using a spectrophotometric plate reader BMG Optima plate reader at 1, 3, 6, 24, 48 and 72 h of treatment. Each cell line was analysed in triplicate on three independent occasions.

### 4.9. Statistical Analysis

Statistical analyses were conducted using both Statistica 10 v.10 (StatSoft, Inc., Tulsa, USA) and Past 3 statistical software [60], with the exception of the survival analyses that was conducted using GraphPad Prism version 6.0.0 for Windows (GraphPad Software Inc, San Diego, USA).

Before statistical analysis, all mitochondrial and cellular functional data were tested for normality using Shapiro-Wilks W and the Kolmogorov-Smirnoff test and log transformed, where appropriate.

Major effects such as differences between cell lines (including with and without F18L) were determined using analysis of variance (ANOVA) for normal data. Individual contrasts between effects within ANOVAs were examined using orthogonal polynomial coefficients. Post hoc comparisons were performed using least significant difference (LSD) tests.

For the multivariate analyses, all mitochondrial respiratory chain enzyme activity and CoQ10 level data were standardised (mean = 0 and SD = 1) to avoid dominance effects between the variables. Principal component analysis was then used in conjunction with multiple regression analysis to examine relationships between the variables, including MRC complex activities of the cell lines and F18L-grouping. Discriminant function analysis was used as a way of testing how well the different cell lines analysed could be categorised into the newly identified MRC activity sub-groups without any other a priori knowledge. Data on individual cells were extracted one by one from the data set and the discriminant function analysis was performed repeatedly (jackknifed) so that each of the cell assignments was made independently of the larger dataset. The percentage of correct predictions was estimated and used as a measure of the overall effectiveness of the multivariate methods. Although it was possible to examine the relationship between MRC complex activities and CoQ10 levels using both PCA and multiple regression analysis, it was not possible to examine how these parameters in combination related to F18L grouping due to too few CoQ10 level data points being available.

The Kaplan-Meier method was to estimate overall survival rates of the patients based on various groupings, and the Log-rank (Mantel-Cox) test was used to compare rates between groups.

## Supplementary Materials

Supplementary materials can be found at https://www.mdpi.com/1422-0067/20/13/3364/s1.
