# Supplementary material for: Integrated Approach Reveals Role of Mitochondrial Germ-Line Mutation F18L in Respiratory Chain, Oxidative Alterations, Drug Sensitivity, and Patient Prognosis in Glioblastoma
Source: Int J Mol Sci. 2019 Jul 9;20(13):3364. doi: 10.3390/ijms20133364 (PMC6651022; doi:10.3390/ijms20133364)
Supplement: Supplementary file 1 [file ijms-20-03364-s001.pdf]

- **Supplementary Information**

Supplementary Materials and Methods

Table S1A Details of cell lines derived from primary adult glioblastoma screened for F18L and used in the mitochondrial and cellular functional studies.

Table S1B Details of TCGA glioblastoma patient and tissues used in the F18L survival studies.

Table S2A Details of differences between F18L positive and negative cells analysed by ANOVA using a regression approach.

Table S2B Details of the discriminant function analysis.

Table S2C Details of PCA approach looking at correlations between individual MRC activities and CoQ10 levels.

Table S3 Details of the oxidative defence genes tested for a role in clomipramine sensitivity.

## 1. Supplementary Materials and Methods

### Establishment and preparation of cell cultures for DNA extraction, and mitochondrial and cell function assays

Biopsies originating from the main tumor mass were verified histologically as GBM and transferred to a sterile petri dish; rinsed with Hank's Balanced Salt Solution and disaggregated to form a uniform cell suspension. This suspension was then transferred into a tissue culture flask containing Dulbecco's Modified Eagle Medium (DMEM) + 20% fetal bovine serum (FBS) for 7 days until cells attached. After establishment, all cell cultures were grown in DMEM + 10% FBS, with the exception of the control cell line which was grown in astrocyte growth medium + 10% human serum, in a humidified atmosphere at 37°C and 5% $CO_2$ and 25% $O_2$ until 80% confluent. The cells were then detached using trypsin, mixed with growth medium and centrifuged at 200 × g to either form a pellet for DNA extraction, or processing for mitochondrial and cell function assays.

Table S1A Details of cell lines derived from primary adult glioblastoma (unless otherwise indicated) screened for F18L and used in the mitochondrial and cellular functional studies*.

| Cell line | | | | | | | Patient | | | | | | |
|---|---|---|---|---|---|---|---|---|---|---|---|---|---|
| **Sample ID** | **Source** | **IDH1 (R132) status** | **IDH2 (R132) status** | **MGMT status** | **F18L** | **F18L screening method** | **Karnofsky Score** | **Ki67 (%)** | **Resection Chemotherapy Radiotherapy** | **Age at diagnosis** | **Time to death (months)** | **Ref** | **Genbank Accession No.** |
| SC1800** | Cerebral cortex, non-neoplastic astrocyte | Negative | Negative | Unmethylated | No | Total DNA + long PCR + mtDNA-seq | n/a | n/a | n/a | n/a | n/a | Lloyd et al. 2015 | KJ735675.1 |
| UP-029 | | Negative | Negative | Methylated | No | Total DNA + long PCR + mtDNA-seq | n.i.a | n.i.a | n.i.a | 66 | n.i.a | Lloyd et al. 2015 | KJ735668.1 |
| SNB-19*** | | Negative | Negative | Methylated | No | Total DNA + long PCR + mtDNA-seq | n.i.a | n.i.a | n.i.a | n.i.a | n.i.a | Lloyd et al. 2015 | KJ735677.1 |
| SEBTA-023 | | Negative | n.i.a | n.i.a**** | Yes | Total DNA + long PCR + mtDNA-seq | n.i.a | 30% | Stupp protocol | 70 | 33.33 | This study | MN150712 |
| UP-007 | | Negative | Negative | Methylated | Yes | Total DNA + long PCR + mtDNA-seq | n.i.a | n.i.a | n.i.a | 71 | n.i.a | This study | MN150713 |

* Passages used for the functional studies: SC1800 (6), UP-029 (19-28), SNB-19 (n.i.a.), SEBTA-023 (11-20), and UP-007 (26)

**ScienCell Research Laboratories

***DSMZ German Collection of Microorganisms and Cell Cultures

****Original tumour was methylated

n.i.a No information available

UP- and SEBTA- prefixes denote cell cultures were obtained in house from biopsies derived from Kings College Hospital (London, UK). All cells were confirmed to be mycoplasma-free and authenticated by short-term tandem repeat-PCR and capillary electrophoresis using an Applied Biosystems 3130xl Genetic Analyzer (profiles available on request).

Table S1B Details of TCGA glioblastoma patient and tissues used in the F18L survival studies.

| PATIENT BARCODE | Subtype | sil_width | CORE_sample | GENDER | VITALSTATUS | KarnScore | AgeAtFirstDiagnosis | Survival (days) | MGMT_methylated | % Tumor Nuclei | % Necrosis | F18L |
|---|---|---|---|---|---|---|---|---|---|---|---|---|
| TCGA-02-0003 | Proneural | 0.21 | CORE | MALE | DEAD | 100.00 | 50.22 | 144.00 | Unmethlyated | 100.00 | 12.50 | No |
| TCGA-02-0033 | Mesenchymal | 0.44 | CORE | MALE | DEAD | 100.00 | 54.95 | 86.00 | Unmethlyated | 100.00 | 15.00 | No |
| TCGA-02-0047 | Proneural | 0.13 | CORE | MALE | DEAD | 90.00 | 78.74 | 448.00 | Unmethlyated | 92.50 | 5.00 | Yes |
| TCGA-02-0055 | Mesenchymal | 0.42 | CORE | FEMALE | DEAD | 90.00 | 62.42 | 76.00 | Unmethlyated | 95.00 | 15.00 | Yes |
| TCGA-06-0122 | Mesenchymal | 0.15 | CORE | FEMALE | DEAD | 70.00 | 84.80 | 181.00 | Unmethlyated | 100.00 | 20.00 | No |
| TCGA-06-0124 | Mesenchymal | 0.26 | CORE | MALE | DEAD | 70.00 | 67.33 | 620.00 | Methylated | 100.00 | 10.00 | Yes |
| TCGA-06-0125 | Classical | 0.23 | CORE | FEMALE | DEAD | 70.00 | 63.91 | 1448.00 | Unmethlyated | 100.00 | 5.00 | No |
| TCGA-06-0126 | Classical | 0.10 | CORE | MALE | DEAD | 70.00 | 86.59 | 211.00 | Unmethlyated | 100.00 | 0.00 | No |
| TCGA-06-0128 | Proneural | 0.15 | CORE | MALE | DEAD | 90.00 | 66.30 | 691.00 | Unmethlyated | 97.50 | 7.50 | No |
| TCGA-06-0129 | Proneural | 0.27 | CORE | MALE | DEAD | 100.00 | 30.89 | 1024.00 | Unmethlyated | 97.50 | 0.00 | No |
| TCGA-06-0130 | Mesenchymal | 0.39 | CORE | MALE | DEAD | 90.00 | 54.34 | 358.00 | Unmethlyated | 80.00 | 5.00 | No |
| TCGA-06-0132 | Neural | 0.18 | CORE | MALE | ALIVE | NA | 49.62 | 295.00 | Unmethlyated | 97.50 | 2.50 | No |
| TCGA-06-0137 | Classical | 0.22 | CORE | FEMALE | DEAD | 90.00 | 63.72 | 812.00 | Unmethlyated | 92.50 | 2.50 | No |
| TCGA-06-0138 | Neural | 0.14 | CORE | MALE | DEAD | 90.00 | 43.08 | 737.00 | Unmethlyated | 97.50 | 2.50 | No |
| TCGA-06-0139 | Mesenchymal | 0.41 | CORE | MALE | DEAD | 60.00 | 40.33 | 362.00 | Unmethlyated | 95.00 | 0.00 | Yes |
| TCGA-06-0145 | Classical | 0.21 | CORE | FEMALE | DEAD | NA | 53.83 | 71.00 | Methylated | 100.00 | 5.00 | No |
| TCGA-06-0152 | Mesenchymal | 0.05 | CORE | MALE | DEAD | NA | 68.03 | 373.00 | Unmethlyated | 97.00 | 36.50 | Yes |
| TCGA-06-0154 | Mesenchymal | 0.21 | CORE | MALE | DEAD | 100.00 | 54.81 | 424.00 | Unmethlyated | 95.00 | 35.00 | No |
| TCGA-06-0156 | Proneural | 0.15 | CORE | MALE | DEAD | NA | 57.39 | 148.00 | Unmethlyated | 97.50 | 5.00 | No |
| TCGA-06-0157 | Classical | -0.02 | NO | FEMALE | DEAD | 50.00 | 63.32 | 97.00 | Unmethlyated | 97.50 | 5.00 | No |
| TCGA-06-0158 | Classical | -0.03 | NO | MALE | DEAD | 80.00 | 73.53 | 161.00 | Unmethlyated | 97.50 | 2.50 | Yes |
| TCGA-06-0166 | Proneural | 0.17 | CORE | MALE | DEAD | NA | 51.75 | 178.00 | Unmethlyated | 92.50 | 30.00 | No |
| TCGA-06-0168 | Mesenchymal | -0.04 | NO | FEMALE | DEAD | 100.00 | 59.62 | 598.00 | Unmethlyated | 92.50 | 30.00 | No |
| TCGA-06-0171 | Neural | 0.12 | CORE | MALE | DEAD | 100.00 | 65.94 | 399.00 | Unmethlyated | 92.50 | 5.00 | No |
| TCGA-06-0173 | Neural | 0.08 | CORE | FEMALE | DEAD | NA | 72.69 | 136.00 | Methylated | 95.00 | 17.50 | Yes |
| TCGA-06-0174 | Proneural | 0.32 | CORE | MALE | DEAD | 80.00 | 54.28 | 98.00 | Methylated | 97.50 | 10.00 | No |
| TCGA-06-0176 | Mesenchymal | 0.32 | CORE | MALE | ALIVE | 90.00 | 34.98 | 954.00 | Unmethlyated | 92.50 | 35.00 | No |
| TCGA-06-0178 | Neural | -0.06 | NO | MALE | ALIVE | NA | 39.03 | 1168.00 | Unmethlyated | 100.00 | 0.00 | No |
| TCGA-06-0184 | Mesenchymal | 0.11 | CORE | MALE | ALIVE | 90.00 | 63.84 | 907.00 | Unmethlyated | 95.00 | 5.00 | No |
| TCGA-06-0185 | Neural | 0.09 | CORE | MALE | ALIVE | 100.00 | 54.55 | 846.00 | Methylated | 100.00 | 5.00 | No |
| TCGA-06-0187 | Classical | 0.07 | CORE | MALE | ALIVE | 70.00 | 69.32 | 414.00 | Methylated | 100.00 | 10.00 | No |

| | | | | | | | | | | | | |
|---|---|---|---|---|---|---|---|---|---|---|---|---|
| TCGA-06-0188 | Neural | -0.10 | NO | MALE | ALIVE | 100.00 | 71.40 | 389.00 | Unmethlyated | 95.00 | 10.00 | No |
| TCGA-06-0189 | Mesenchymal | 0.35 | CORE | MALE | DEAD | NA | 55.57 | 468.00 | Unmethlyated | 100.00 | 32.50 | Yes |
| TCGA-06-0190 | Mesenchymal | 0.35 | CORE | MALE | DEAD | 80.00 | 62.52 | 317.00 | Unmethlyated | 100.00 | 40.00 | No |
| TCGA-06-0195 | Neural | 0.09 | CORE | MALE | DEAD | 100.00 | 63.33 | 225.00 | Methylated | 90.00 | 0.00 | No |
| TCGA-06-0210 | Mesenchymal | 0.21 | CORE | FEMALE | DEAD | NA | 72.83 | 225.00 | Unmethlyated | 92.50 | 10.00 | Yes |
| TCGA-06-0211 | Classical | 0.15 | CORE | MALE | DEAD | 100.00 | 47.95 | 360.00 | Unmethlyated | 92.50 | 0.00 | No |
| TCGA-06-0214 | Neural | 0.16 | CORE | MALE | DEAD | 40.00 | 66.22 | 457.00 | Methylated | 92.50 | 7.50 | No |
| TCGA-06-0219 | Neural | 0.27 | CORE | MALE | DEAD | 40.00 | 67.07 | 15.00 | Unmethlyated | 100.00 | 12.50 | No |
| TCGA-06-0221 | Neural | 0.05 | CORE | MALE | DEAD | 40.00 | 31.03 | 603.00 | Methylated | 90.00 | 5.00 | Yes |
| TCGA-06-0237 | Neural | 0.20 | CORE | FEMALE | DEAD | 40.00 | 75.94 | 415.00 | Unmethlyated | 100.00 | 5.00 | No |
| TCGA-06-0238 | Proneural | 0.11 | CORE | MALE | DEAD | NA | 46.65 | 405.00 | Unmethlyated | 100.00 | 5.00 | No |
| TCGA-06-0241 | Proneural | 0.31 | CORE | FEMALE | ALIVE | 40.00 | 65.99 | 198.00 | Methylated | 100.00 | 7.50 | No |
| TCGA-06-0644 | Mesenchymal | 0.34 | CORE | MALE | ALIVE | 80.00 | 71.86 | 122.00 | Unmethlyated | 95.00 | 20.00 | No |
| TCGA-06-0645 | Mesenchymal | 0.32 | CORE | FEMALE | ALIVE | NA | 55.99 | 98.00 | Methylated | 97.50 | 15.00 | No |
| TCGA-06-0646 | Proneural | 0.05 | CORE | MALE | DEAD | 90.00 | 60.98 | 175.00 | Unmethlyated | 95.00 | 7.50 | No |
| TCGA-06-0648 | Proneural | 0.36 | CORE | MALE | ALIVE | 90.00 | 77.97 | 77.00 | Methylated | 100.00 | 10.00 | No |
| TCGA-08-0386 | Neural | 0.02 | CORE | MALE | DEAD | 90.00 | 74.07 | 548.00 | Methylated | 97.50 | 7.50 | No |
| TCGA-12-0615 | Classical | -0.03 | NO | FEMALE | DEAD | 70.00 | 78.26 | 467.00 | Unmethlyated | 100.00 | 25.00 | Yes |
| TCGA-12-0616 | Proneural | 0.22 | CORE | FEMALE | DEAD | 100.00 | 36.83 | 448.00 | Unmethlyated | 100.00 | 27.50 | No |
| TCGA-12-0618 | Proneural | 0.32 | CORE | MALE | DEAD | 70.00 | 49.48 | 395.00 | Unmethlyated | 100.00 | 6.50 | No |
| TCGA-12-0619 | Mesenchymal | 0.27 | CORE | MALE | DEAD | 90.00 | 60.01 | 1062.00 | Methylated | 100.00 | 7.50 | No |

The TCGA glioblastoma samples were originally classified into subtypes by Verhaak et al. 2010. From the original study that included 202 samples, only adult patients with primary GBM where we could also determine F18L status were included.

Table S2A Details of differences between F18L positive and negative cells analysed by ANOVA using a regression approach.

| Test of SS Whole Model vs. SS Residual (Spreadsheet of adult cells only) | | | | | | | | | | | |
|---|---|---|---|---|---|---|---|---|---|---|---|
| | **Multiple - R** | **Multiple - $R^2$** | **Adjusted - $R^2$** | **SS - Model** | **df - Model** | **MS - Model** | **SS - Residual** | **df - Residual** | **MS - Residual** | **F** | **p** |
| CI activity/CS | 0.51 | 0.26 | 0.24 | 10.84 | 1.00 | 10.84 | 30.79 | 34.00 | 0.91 | 11.98 | 0.001472 |
| CII activity/CS | 0.51 | 0.26 | 0.23 | 10.68 | 1.00 | 10.68 | 31.07 | 34.00 | 0.91 | 11.69 | 0.001652 |
| CII+III activity/CS | 0.69 | 0.47 | 0.45 | 19.91 | 1.00 | 19.91 | 22.44 | 34.00 | 0.66 | 30.17 | 0.000004 |
| CIV activity/CS | 0.46 | 0.21 | 0.19 | 5.89 | 1.00 | 5.89 | 22.08 | 34.00 | 0.65 | 9.07 | 0.004886 |

Table S2B Details of the discriminant function analysis.

| Point | Given group | Classification | Jackknifed |
|---|---|---|---|
| 3 | UP007 | UP007 | UP007 |
| 4 | UP007 | UP029 | UP029 |
| 5 | UP007 | UP007 | UP007 |
| 6 | UP029 | UP029 | UP029 |
| 7 | UP029 | UP029 | UP029 |
| 8 | UP029 | UP029 | UP029 |
| 9 | Sebta 023 | Sebta 023 | Sebta 023 |
| 10 | Sebta 023 | Sebta 023 | Sebta 023 |
| 11 | Sebta 023 | Sebta 023 | Sebta 023 |
| 12 | SNB19 | SNB19 | SNB19 |
| 13 | SNB19 | SNB19 | SNB19 |
| 14 | SNB19 | SNB19 | SNB19 |
| 15 | UP007 | UP007 | UP007 |
| 16 | UP007 | UP007 | UP007 |
| 17 | UP007 | UP007 | UP007 |
| 18 | UP029 | UP029 | UP029 |
| 19 | UP029 | UP007 | UP007 |
| 20 | UP029 | UP007 | UP007 |
| 21 | Sebta 023 | Sebta 023 | Sebta 023 |
| 22 | Sebta 023 | Sebta 023 | Sebta 023 |
| 23 | Sebta 023 | Sebta 023 | Sebta 023 |
| 24 | SNB19 | SNB19 | SNB19 |
| 25 | SNB19 | SNB19 | UP007 |
| 26 | SNB19 | SNB19 | SNB19 |
| 27 | UP007 | UP007 | UP007 |
| 28 | UP007 | UP007 | UP007 |
| 29 | UP007 | UP007 | UP007 |
| 30 | UP029 | UP029 | SNB19 |
| 31 | UP029 | UP029 | UP029 |
| 32 | UP029 | UP029 | UP029 |
| 33 | Sebta 023 | Sebta 023 | Sebta 023 |

Green = correctly classified

Red = incorrectly classified

| | | | |
|---|---|---|---|
| 34 | Sebta 023 | Sebta 023 | Sebta 023 |
| 35 | Sebta 023 | Sebta 023 | Sebta 023 |
| 36 | SNB19 | SNB19 | SNB19 |
| 37 | SNB19 | SNB19 | SNB19 |
| 38 | SNB19 | SNB19 | SNB19 |

Table S2C Details of PCA approach looking at correlations between individual MRC activities and CoQ10 levels.

| | CI/CS activity | CII/CS activity | CIV/CS activity | CII+III/CS activity | CoQ10/CS |
|---|---|---|---|---|---|
| **CI/CS activity** | 1 | 0.1924 | 0.0034 | -0.3477 | 0.7763 |
| | p= --- | p=.549 | p=.992 | p=.268 | p=.003 |
| **CII/CS activity** | 0.1924 | 1 | 0.5799 | 0.5382 | 0.0552 |
| | p=.549 | p= --- | p=.048 | p=.071 | p=.865 |
| **CIV/CS activity** | 0.0034 | 0.5799 | 1 | 0.642 | -0.1498 |
| | p=.992 | p=.048 | p= --- | p=.024 | p=.642 |
| **CII+III/CS activity:** | -0.3477 | 0.5382 | 0.642 | 1 | -0.5815 |
| | p=.268 | p=.071 | p=.024 | p= --- | p=.047 |
| **CoQ10/CS** | 0.7763 | 0.0552 | -0.1498 | -0.5815 | 1 |
| | p=.003 | p=.865 | p=.642 | p=.047 | p= --- |

Values are expressed as correlation coefficients (r values) and these are associated and presented with their relevant significances (p values). Significant correlations are highlighted in red.

Table S3 Details of the oxidative defence genes tested for a role in clomipramine sensitivity.

| Gene | Function in oxidative stress defense | Localisation |
|---|---|---|
| Prx1 | Thioredoxin peroxidase | Mito |
| Ahp1 | Thioredoxin peroxidase | Cyto |
| Tsa1 | Thioredoxin peroxidase | Cyto |
| Tsa2 | Thioredoxin peroxidase | Cyto |
| Dot5 | Thioredoxin peroxidase | nucleus |
| Ccp1 | Cytochrome c peroxidase | MIS |
| Aim32 | Thioredoxin like protein | mito |
| Gtt1 | Glutathione peroxidase | ER |
| Grx1 | Glutaredoxin | cyto/nucleus |
| Grx2 | Glutaredoxin | cyto/mito |
| Fms1 | Polyamine oxidase | cyto |
| Yhb1 | Nitric oxide oxidoreductase | cyto/ mito |
| Mcr1 | Cytochrome-b5 reductase | MIS |
| Cta1 | Catalase | mito |
| Ctt1 | Catalase | cyto |
| Sod1 | Superoxide dismutase 1 | cyto/mito |
| Sod2 | Superoxide dismutase 2 | mito |
